# The impact of the pandemic on psychophysical well-being and quality of learning in the growth of adolescents (aged 11–13): a systematic review of the literature with a PRISMA method

**DOI:** 10.3389/fpsyg.2024.1384388

**Published:** 2024-09-19

**Authors:** Marta Schiera, Fabiola Faraci, Giuseppe Mannino, Lorenzo Vantaggiato

**Affiliations:** ^1^Libera Università Maria SS. Assunta, Rome, Italy; ^2^Gonzaga International School, Palermo, Italy

**Keywords:** COVID-19, adolescents, students, middle school, wellbeing, learning

## Abstract

**Introduction:**

This study examines the social impact and psychological effects of the COVID-19 pandemic on the growth of adolescents students, aged 11–13, on well-being and learning.

**Methods:**

Said review, therefore, will consider all the report of scientific works published since 2020 on the following platforms: SCOPUS, PsycoInfo, and Web of Science. Specifically, the research has selected all studies concerning students aged 11–13. Furthermore, the authors have restricted the scope of the study to two fundamental aspects of the above-mentioned subjects, such as their physical and psychological well-being and didactic learning following the COVID-19 pandemic. The justification for choosing such themes lies in the fact that the SARS-CoV-2 pandemic and the ensuing measures adopted to curb its spread have had -and very likely will continue having- sizable repercussions in the life of each individual, especially in students considered, whose psychological and physical well-being has been considerably affected.

**Results:**

In particular, during the peak of the pandemic and its subsequent phases, the various measures issued to limit contagion have completely compromised and disregarded the rights of children and youngsters. Suddenly, leisure, sport, play, relations, and socialization disappeared from their lives. Besides, in the early stages of the pandemic, remote teaching replaced face-to-face teaching, later to become an intermittent system in the red- and orange-labelled areas of the country. Consequently, such measures have severely limited opportunities for learning, growth, curiosity and creating relationships that are typical of both a school and outside environment. Concerning the well-being of the subjects considered, a review of the target literature indicates a wide range of psychological and physical disorders, such as malaise, eating and sleep disorders, addiction to games, internet, alcohol, and drugs. For what regards, instead, the realm of learning, most studies highlight a diminished school performance as the main implication of remote teaching. Furthermore, a remarkable number of studies reveals substantial social inequalities; specifically, students coming from middle class families have managed to maintain higher quality educational standards in a critical context such as the pandemic, while those students coming from socially disadvantaged families have had fewer learning opportunities both in terms of time and range of experiences (due to lack of electronic devices, Internet connectivity, homework, continuity of after-school activities). In summary, if the school system pre-COVID-19 already presented significant limitations in its ability to reduce existing social inequalities, school closures during lockdowns pose enormous challenges in developing effective policies to compensate learning losses and inequalities.

**Discussion:**

Therefore, based on the results shown, the necessity arises of an emergency plan with social and educational objectives to restore those social, emotional, and cognitive abilities that are compromised. In general, it is the responsibility of countries and governments to guarantee the right to education in these difficult times, while avoiding any type of inequalities and establishing a digital learning system, that allows to face situations such as those generated by the Coronavirus, thus preventing those situations of serious anxiety and stress that have affected most subjects.

## Introduction

The aim of this work is to systematically review the research that has studied and analyzed the psychophysical effects of the Covid-19 pandemic on the growth of young students (ages 11–13 years), in terms of psychophysical well-being and learning quality, as well as on the implications that the measures adopted to counter the emergency have determined both in terms of mental and physical health (psychophysical well-being) in the subjects studied, both in terms of learning quality.

Psychophysical well-being and learning quality are described through the stages of development of personal autonomy and peer study and socialization. They are also interconnected.

The pandemic has very negatively affected their mental health and led to an increase in emotional disorders, eating and sleep disorders, anxiety, and depression (Cooper et al., 2021; Cenat et al., 2022; Ludwig-Walz et al., 2022). Moreover, recent studies have highlighted a decrease in the age of consumption of heavy drugs below 14 (Villanti et al., 2022; Herzig et al., 2023).

The choice of examining subjects aged 11–13 is not random. In fact, considering their age, these are the main actors in the process of growth and thus more sensitive to sudden events, such as a pandemic.

Adolescence in the first stage (11–13) is a fundamental part of development in the life of a future adult, characterized by several moments of stress (Casey et al., 2010). This is due to the physical, mental, chemical and social changes that the subject goes through in this phase in which the self-regulation system plays a fundamental role, because it allows the subject to learn to manage and control his impulses, to regulate emotions, to modulate behaviors, to respond effectively to environmental demands (Somerville, 2013).

Another typical feature of this early adolescent phase is the marked increase in social sensitivity and the importance of relationships with peers (Somerville, 2013). In fact, adolescents begin to experience their autonomy from the family group and begin to fight for their independence, getting closer and closer to the peer group. Peers become, therefore, the main source of interaction and influence (Meuwese et al., 2017).

If, in fact, in early childhood, a foundational role in the dynamics of learning is given by the family, both in the positive cases of formation of free, open and dialogic thought and in the negative cases of formation of saturated, closed and prejudicial thought (Mannino and Giunta, 2015); from the very early stages of adolescence and up to young adulthood, an increasing role is assumed by the school and the peer group (Mannino et al., 2021).

The uprooting of daily life, social isolation, uncertainty regarding the future, mixed with the fear of contracting the virus for themselves and their dear ones, have provoked a series of effects on young people’s mental health. The sudden disappearance of all those activities that conveyed a sense of daily routine, such as school, university, meeting places caused and worsened various psychological disorders. Indeed, digital devices (PCs, tablets, smartphones) represented the only “window onto the world,” especially during the lockdown stage. Electronic means became the only instruments for conducting virtually every activity, from remote learning to leisure and socialization (Mannino et al., 2017). This implied a 50% increase in the use of the Internet during the spread of COVID-19, therefore heightening dependence on such technologies (Iacolino et al., 2019; UNICEF, 2020).

The spread of the pandemic and the ensuing remote teaching have generated a so-called learning loss, i.e., a serious drop in students’ competence levels caused by the long-lasting disruption of learning trajectories (Yan et al., 2021; Valido et al., 2023). In fact, the school represents the main place where, through dialogue and discussion, every individual forms their identity and personality. Therefore, it is evident how the school environment does not exclusively coincide with the mere teaching, but represents a vital space for a healthy and consistent development -both physical and psychological- of children and adolescents. What characterizes the school environment is a set of diverse and important values, such as relationships with peers, discussion, competition, collaboration, engagement, conflict, trust. All these factors concur to establishing a person’s identity.

Contrary to this, the sudden lack of relations and social isolation caused by lockdowns have implied a real upheaval in the lives of younger people, with inevitable, alarming psychological repercussions. Young people have suddenly felt alone, abandoned, lost, and locked in forced solitude. Such a radical change led them to live an extremely challenging situation for their growth, psychological and physical well-being (Bernasco et al., 2021; An et al., 2022).

In light of this, this study means to suggest that the Ministry of Education and other stakeholders consider introducing digital remote teaching as an alternative educational approach, to encourage students and teachers to become more competent in the use of technologies, while improving their cognitive abilities. Therefore, it is necessary to configure a digital learning system that guarantees adequate, efficient education in view of future pandemics or other events that can disrupt the country’s educational system.

## Methodology

Without claiming to be exhaustive, the above-mentioned systematic review of the literature means to explore the psychological effects -in connection with physical, and learning ones- of the social impacts caused by covid-19 on the growth of adolescents (aged 11–13), according to the criteria and objectives indicated in the previous paragraph.

Said systematic review, therefore, aims to answer the following questions: What is the state of the art in COVID-19-related studies on adolescents from 2020 to today? What are the most prominent themes in COVID-19-related studies on adolescence from a psychophysical viewpoint on wellbeing and learning? What are some suggestions for future research?

### Criteria for inclusion and exclusion

Based on the objectives of revision and the questions that have guided the research on documents, a revision protocol has been developed to guide the research on the literature. The revision protocol contains databases, keywords and criteria for inclusion and exclusion of the literature.

With regards to inclusion criteria to determine the suitability of the articles for the realization of said revision, the authors have only considered studies meeting the following criteria: (a) participants must be adolescents aged 11–13 (comprising only those works specifically considering that age range or including it); (b) results must highlight the psychological effects -connected with physical, on learning generated by the CoViD-19 pandemic; (c) non-randomized quantitative studies (e.g., case and control studies), quantitative descriptive studies (e.g., transversal studies, longitudinal studies to better comprehend short- and long-term effects), mixed methods (i.e., qualitative and quantitative) or qualitative methods; (d) studies must have been conducted during the various phases of the CoViD-19 pandemic (i.e., during the first and/or second wave and/or final stage).

Non-eligible, excluded studies comprise: (a) studies not specifically focusing on the psychological effects -connected with physical, on learning- generated by the CoViD-19 pandemic on groups of adolescents; (b) studies not conducted during the COVID-19 pandemic or not specifying the exact timeframe of the research; (c) studies that do not clearly and patently specify the age range of the sample considered; (d) studies including clinical subjects; (e) letters to editors, comments or studies that do not detail research protocols.

### Keywords and selection of works

In the selection of studies, the authors have utilized the following keywords to explore the psychological effects and the social impact of the pandemic on adolescents aged 11–13: “CoViD-19 AND middle school”; “CoViD-19 AND learning”; “CoViD-19 AND early adolescents”; “CoViD-19 AND well-being.”

We therefore wanted to look for all the scientific literature that in the years indicated covered the area of interest of middle school and that showed research on the quality of learning, psychophysical repercussions and general well-being of adolescents (11–13 years).

To afford a multidisciplinary scope, the relevant literature comes from the most significant electronic data banks that could help authors find works in a psychological, and educational realm: SCOPUS, PsycoInfo, Web of Science. Whenever the literature was not accessible through the above-mentioned data banks, the Google Scholar search engine represented an alternative option.

The terms used for the literature search appear in the title, in the keywords, in the abstract or in the main body of the articles. Furthermore, the search has considered all those documents (both national and international articles) in academic journals in English between 2020 and 2023. As the English language is predominant in international academic publications, this is the principal reason why we have chosen academic reviews published in English that cover the chosen timeframe, as this spans the years when the CoViD-19 pandemic was declared.

### Evaluation of the quality of studies

For the identification and subsequent selection of the studies, in addition to the decision and insertion of keywords, the researchers used the Consensual Quality Research (CQR) method. This methodology helps to overcome any underlying bias or biased view, which may compromise the aforementioned systematic study. This is a specific methodology for social research, which allows to monitor and control any semantic or value distortion expressed by the individual researcher through the report and the circular group discussion, mediated by a specific observation grid.

Consensual Quality Research (CQR) finds its definition and description in a paper by Hill et al. (2005), although it had already been introduced by some of them (Hill et al., 1997) in 1997, with the aim of bringing together various qualitative research techniques in the form of a rigorous and easy-to-apply method.

Each researcher was instructed in the method. A working grid has been set up to evaluate the methodological quality of each individual study according to common criteria through some indicators. These indicators were chosen and entered collectively and the grid was shared digitally (included in this paper). Each researcher participated in the decision and insertion of the keywords in the search engine (scopus) and had assigned a part of the search records (second progressive step of the list in alphabetical order) and expressed his evaluation for each individual paper, on the specific shared grid.

Every week for a period of 2 months, sharing and group evaluation sessions were carried out to arrive at a single and definitive grid.

Each article was therefore selected and evaluated in two phases, by the individual researcher and by the group, with reference to a single grid.

#### Screening methodology for the literature

We have chosen to use the Preferred Reporting Items for Systematic Reviews (PRISMA) methodology for the screening of the literature and for compiling our review (PRISMA, 2019b). The PRISMA (2019a) checklist establishes the necessary steps to implement a review that can be replicated by other scholars and generate reliable data.

Therefore, we believe that a systematic review aligned with the PRISMA checklist would better convey the execution, the quality, and the rigor we have pursued in realizing this systematic review of the literature. Moreover, this methodology would broaden the potential of existing reviews on the matter at hand (Loades et al., 2020; Lee Y. et al., 2021; O’Reilly et al., 2021), thus enriching the results exposed in the literature.

For this reason, the authors preferred the method of systematic review, excluding the meta-analysis procedure, as the objective was to understand the state of the research on the subject, in order to plan and design, at a later time, an updated study in the field.

The literature search began on 13 June 2023 and ended on 21/07/2023, with a total of 156 studies found.

Five researchers performed the ensuing selection engaging in constant dialogue and discussion to limit the risk of errors and incorrect exclusions with the CQR methodology (Hill et al., 2005). In fact, the selected scientific documents only include those works that study the impact on psychophysical well-being and social impacts caused by remote teaching on the learning of adolescents, who have experienced the CoViD-19 pandemic.

The first phase of the screening process has excluded 23 editorials, proceedings of conferences, book chapters and reviews; next, 7 duplicated studies have been removed.

The remaining 126 studies were screened according to the above-mentioned selection criteria. Due to the difficulty in finding research among the documents examined that explicitly and patently indicated the age range of interest for the review (11–13 years), a further 45 articles were excluded, to arrive at a total of 81 studies.

In the final eligibility phase, these remaining 81 studies were carefully examined one by one, based on the selection criteria. If the abstracts did not contain sufficient information, the complete articles were scanned based on the selection criteria.

**Figure 1:**
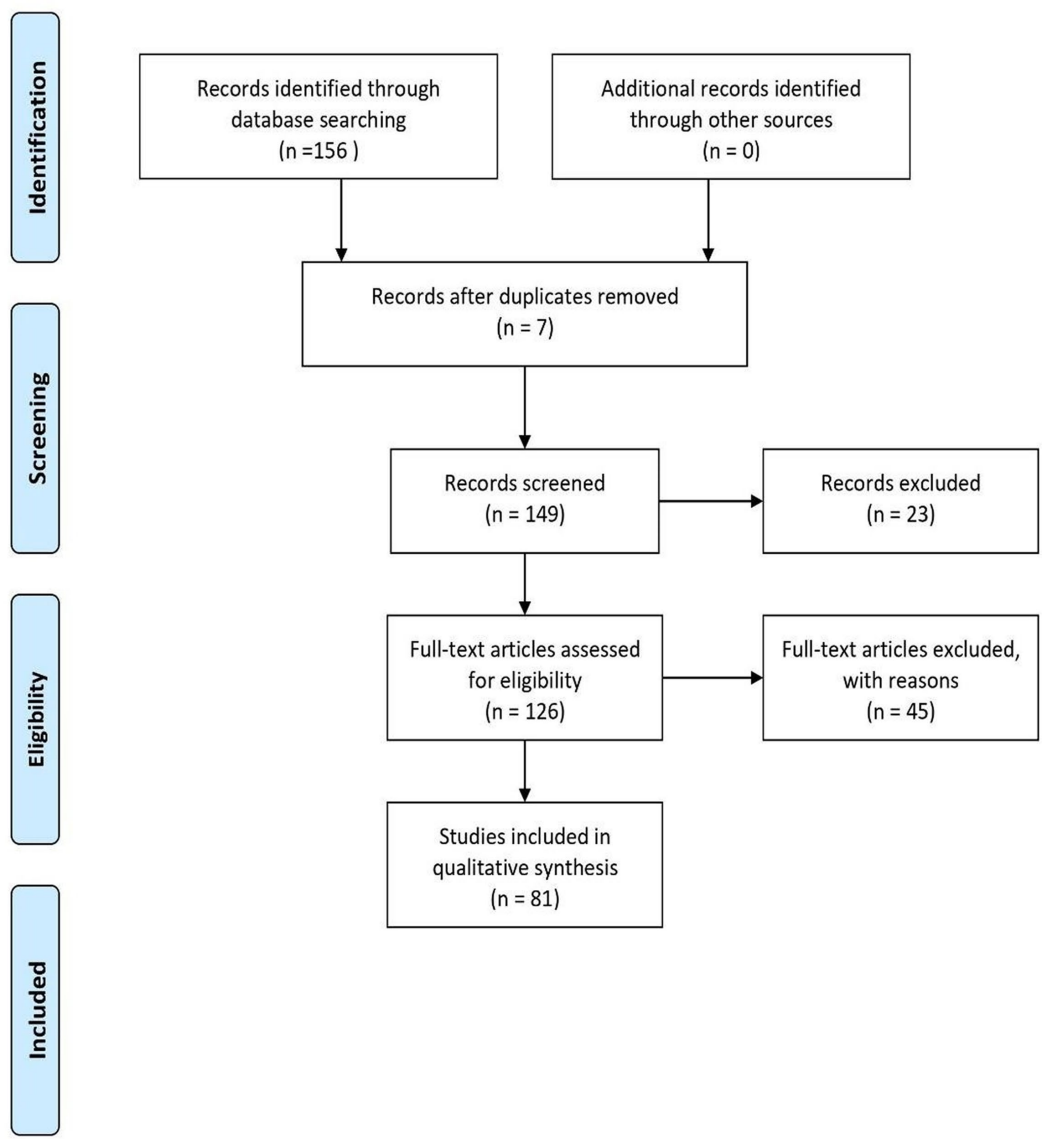
A PRISMA flowchart for literature screening process. Source: Adapted from Moher et al. (2009).

After completing the various analytical and screening phases, these 81 studies were considered as eligible and therefore selected for the final execution of the systematic review of the literature. Figure 1 illustrates the selection process.

### Research review

The international research of the last few years highlights a high incidence of mental health conditions (McQuaid et al., 2021) in the youngest population.

The data show an increase in symptoms of depression, anxiety, psychological malaise, disaffection, mental disorders, and an increase in loneliness (Cooper et al., 2021; Liu et al., 2021) especially in the early and late adolescence (Asscheman et al., 2021).

To these, one must add at-home isolation and social distancing (Loades et al., 2020), lack of physical exercise (Mittal et al., 2020), strain on family relationships (Meade, 2021) and an increase in screen time (Duan et al., 2020).

Cheong et al. (2023) explain that adolescents subjected to high levels of stress due to Covid-19-related worries incurred a higher likelihood of developing mental health disorders (Roche et al., 2022), such as emotional stress (fear, anxiety, depression, neurasthenia) and loneliness. Such difficulties appear to have remained unchanged when the spread settled and even after students returned to school, with the easing of all restrictions (Liu et al., 2021; Zhou et al., 2023). Such data have found further confirmation in the questionnaires administered to parents as well. In fact, parents have confirmed their children’s marked psychological malaise, especially in the form of sleep disorders (Zhao et al., 2022), attention deficit (Delvecchio et al., 2022) and difficulty in managing and regulating emotions (Comelli et al., 2021; Huang et al., 2021; Xin et al., 2022). Orgilés et al.’s (2020) study also reinforces parents’ negative opinion, as they noticed that their children appeared to be less engaged in completing homework and less interested and curious to learn new contents.

By examining the evolution of the mental health disorders that arose during the COVID-19 pandemic, scholars have found a peak of such disorders in the period when schools were closed and social isolation ensued (Capurso et al., 2022; Villanti et al., 2022). Therefore, it is possible to affirm that during lockdowns adolescents have found themselves facing a range of challenging scenarios that have heightened their emotional and behavioral vulnerability (Layman et al., 2023), and as a result caused a strong psychological impact on the psychological and emotional well-being of this sector of the population (Borualogo and Casas, 2022). In turn, this has increased the already high incidence of mental health disorders recorded before the pandemic (Sadler et al., 2021).

In a school context, the COVID-19 pandemic has ushered a second technological era and exposed younger students to screens. In fact, thanks to the internet concrete and tangible aspects of reality have become virtual. One of these are schools.

Although noble, the objective of this strategy has generated widespread malaise in students (Dias et al., 2022) of all ages, who have started losing focus and motivation to study (Kirsch and Vaiouli, 2023), as well as a sense of daily routine. In fact, living through a stressful situation has inevitably led most young people to suffer from medium- and long-term psychological consequences (Balayar and Langlais, 2022), as with a stop to all activities adolescents have modified their natural way of living and acting: they no longer wake up to the sound of an alarm (Weingart et al., 2021), they no longer dress adequately and often neglect personal hygiene or take care of it obsessively, they do not leave their homes to go to school, they do not take public or private transport, they do not interact with peers or strangers and do not organize the rest of their day around social or sports activities. Such a lifestyle has generated feelings of sadness, apathy, fear, uncertainty and frustration, poor impulse control, anxiety, routine alterations, insomnia, hypervigilance, and lack of concentration (Kumar and Nayar, 2020).

Beside the above-mentioned psychological and physical issues (Jin et al., 2022), mostly connected to home lockdown, throughout the pandemic adolescents have manifested great concern over school dynamics, as during the various waves distance learning has been one of the first political actions taken by governments, as schools were deemed to be prime places for contagion.

Indeed, research has found that exposure to changes in daily life have generated higher worry and an increase in depressive symptoms (Tao et al., 2023), panic, somatic symptoms and generalize anxiety (Simoës-Perlant et al., 2022). Multivariate analysis suggests that school-related concerns, including sitting through lessons, managing homework and low-quality online lessons, were univocally associated to an increase in depressive symptoms (Hawes et al., 2022; Liu et al., 2022). These findings are confirmed in an Indonesian study, whereby 60% of students have experienced mental health conditions due to social restrictions and online learning (Julianto et al., 2023). According to Han et al. (2023), said symptoms were instead less serious in those subjects with a higher degree of self-reliance and ability to cope focused on the problem at hand. In fact, a negative coping style would bear negative repercussions on mental health.

Furthermore, Jin et al. (2022) state that remote teaching has had a direct impact on the quality of teaching and learning, as they have explored teachers’ perceptions of the concept of online learning. Such perceptions stem from a qualitative analysis. Furthermore, Jin et al. (2022) report that teachers have harshly criticized online teaching, as it allowed neither face-to-face communication nor prompt feedback from students, thus making it impossible for teachers to keep track of students’ understanding in relation to the content covered in a school day. Moreover, teachers have reported students’ learning status was in strong decline and ineffective, unlike with face-to-face teaching.

From a motivational point of view, some studies argue for learning losses during lockdown (Doz, 2021; Feng et al., 2021; Davidovitch et al., 2022; Toker, 2022; Bertoletti et al., 2023; Borgonovi and Ferrara, 2023; Doz and Doz, 2023). This is confirmed by a longitudinal study by Boruchowicz et al. (2021) on a group of young people aged 12–18. Through data obtained from a national statistical office, these authors have remarked that students during the pandemic have overall dedicated 30% less time to studies compared to before. Further studies confirm that students struggled to comprehend online lessons and showed resistance towards involvement in daily school activities (Khlaif et al., 2021; Kirsch and Vaiouli, 2023). In fact, several studies highlight a preference by adolescents for face-to-face learning, as this allows more interaction and a consistent exchange of feedback between pupils and teachers (Ionescu et al., 2020; Lee J. et al., 2021; Swords et al., 2021; Aguilar et al., 2022).

Regarding the breakdown of the relationship between students and schools, experts have expressed concern for young people’s mental health (Yan et al., 2021; Valido et al., 2023), as schools represent an environment of growth and learning, where individuals interact with other significant adults and peers. Such interaction fosters development of their sense of belonging and involvement in learning, something that was cut short by social restrictions.

A study by Perkins et al. (2021) seeks to highlight how the connection with school (Swartz and Benz, 2022) is associated to adolescents’ mental health, especially when related to social connection, i.e., the relationship with peers (Bernasco et al., 2021; An et al., 2022). By examining the changes in adolescents’ mental health over time and studying students’ relationship with school and peers before and after the pandemic, Perkins et al. (2021) have noticed that most students had initially lived school closures with a positive attitude. Later, the lasting home confinement has produced sentiments of uncertainty and loneliness, as adolescents felt little connection with school, even experiencing high levels of anxiety about school (Lane et al., 2021) and social life. This was the case even after the easing of restrictions and the return to the classroom (Alivernini et al., 2020).

Furthermore, school closures have had a direct impact on more vulnerable young people, i.e., those coming from non-urban, less developed areas. Although more than two thirds of countries have introduced a platform for distance learning, such programs have met with limited success in less developed countries. In fact, almost 30% of these countries was not capable of managing such platforms. Such a data was recorded even before the pandemic, possibly leading only to a worse outcome during the COVID-19 pandemic (ONU, 2020). Rivenbark et al. (2020) confirm that adolescents living in economic and social hardship were more likely, compared to peers growing in more equipped environments, to depend on institutional structures for the resources needed to face the pandemic. Some studies have also demonstrated that economic stress was correlated to high levels of mental health conditions (anxiety and depression).

With reference to social and urban provenance, it is still necessary to consider the difficulties in access to online resources that some students have experienced during the pandemic (Conto et al., 2021; Raby et al., 2021; Stefanidou and Mandrikas, 2023).

Despite efforts by schools to maintain learning activities during lockdown, inequalities in academic learning according to family background (income, level of education) and school (level of education, school sector) have impaired the well-being of students (Akbari, 2021). Therefore, the lack of schooling has annulled the benefits of socialization and interrupted those processes of orientation and accompaniment which are particularly important for adolescent students in the transitions between study, work, and life. Furthermore, the digital divide and the evident differences in access to technological devices has caused some students to have no possibility of connecting to online lessons (Haser et al., 2022) for 3–6 months. Such limitations have become more acute when schools have reduced support and students have asked for more independent work (Bonal and Gonzalez, 2020).

Restrictions and lockdowns have limited people’s daily outings, thus exposing adolescents to a frequent and excessive use of the internet, both for school work and leisure activities.

The Internet thus became a prime instrument for entertainment and the only tool for connection among peers. This has determined a further impact on adolescents’ mental health (Kim et al., 2023), together with a higher risk of Internet (Zhang et al., 2022) and cellphone (Hallauer et al., 2021; Shen et al., 2021) addiction. Lin (2020) has studied Internet addiction in 1060 students in junior high school and discovered that the rate of Internet addiction was 24,4% higher compared to same-age samples before the pandemic. Similarly, in a sample of 2050 children and adolescents, Dong et al. (2020) have discovered that the rate of Internet addiction grew with the development of the pandemic.

Therefore, being the only space where frequenting peers was possible, the online world became a meeting place for many, even though adolescents often experienced moments of victimization on the Internet (Bravo-Sanzana et al., 2022; Ye et al., 2022), which increased the risks of developing mental health issues (Garthe et al., 2023).

The above-mentioned variables are influenced by isolation from peers, by the extended family, by the context of school and after school activities (Eales et al., 2021) which, after being initially lived with less concern, as home confinement continued have caused reactions of angst and sadness, moods of depression and feelings of fear, worry, anger, and anxiety (contracting the illness or losing friendships; Akdogan and Ergin, 2022; Pillay, 2023). Such thoughts, which have grown stronger in a context of home confinement, have in turn determined secondary consequences such as irregular sleep patterns, (Li et al., 2022), lower motivation, higher abulia and feelings of discouragement (Montreuil et al., 2023). This shows how incredibly difficult it has been for adolescents to withstand such stress, whose consequences cannot be underestimated, as a risk exists of raising vulnerable young adults, uncapable of establishing relations and dialectic with the surrounding environment.

Although most research states that the consequences deriving from closure and isolation have caused negative effects on the mental and physical health of adolescents, quite a few studies have also investigated that segment of the population which declared to have positively experienced the COVID-19 pandemic (Vira and Skoog, 2021) and to have perceived an improvement in their health during the first lockdown period (Schmiedeberg and Thonnissen, 2021; Ashworth et al., 2022).

Studies conducted in this area report that the pandemic has led to an improvement in emotional health, because the closure of businesses and confinement at home has allowed much more time to be spent with the family (Gray, 2020; Saini et al., 2023), to improve and increase moments of relaxation and experience academic (Pirrone et al., 2022) and extracurricular (Stone et al., 2021; Silk et al., 2022) commitments with less stress. In other cases, the Covid-19 pandemic has generated new positive scenarios, as highlighted by the study by Anyika et al. (2021). These authors believe in the importance of institutionalizing online distance education or web-based learning system in the post-COVID-19 Nigerian education system, as the study identified that students’ engagement in e-education has increased their competence and confidence in acquiring digital knowledge and skills. Additionally, teachers report that technology-based teaching fosters creativity and immense digital literacy to effectively educate students and enhance skill-based learning.

For adolescents, this global event occurred during a delicate period of their development, exactly when young people become less dependent on their families, strengthen their relationships with peers and significant brain maturation occurs. Therefore, social and emotional support from the context of belonging mitigates the probability of developing risky behaviors (Cooper et al., 2021; Xia et al., 2022).

In light of all these aspects, it is necessary to generate preventive and supportive alliances, not only to limit the consequences resulting from the proliferation of the virus, but to contribute to the regeneration of the ecosystem, of the rules of the game, of being together. For example, in studying adolescents’ resilience to COVID-19 pandemic stress and examining key aspects of well-being, Shukla et al. (2022) sought to understand how British, Israeli, and Indian networks have worked to combat negative outcomes of the pandemic. These authors could see that the British and Israeli networks focused on “dealing with problems well,” while the Indian network focused on “feeling useful.” This may have fostered recovery from part of the negative effects generated by the COVID-19 pandemic.

Certainly, on such a large-scale problem, any intervention project must start within the social environment. Within this context it becomes a priority to include aid professionals who, thanks to their skills, know how to adequately manage the effects deriving from this pandemic, in a more effective and targeted way. In fact, their knowledge and experience are used to monitor the situation and to coordinate support measures in favor of all those who need support from both a social and psychological point of view (Salmela-Aro et al., 2021; Schwartz et al., 2021).

## Discussion

The field of investigation concerning the psychological effects and social impact of the Covid-19 pandemic has progressively expanded. In fact, numerous studies have emerged in the literature that seek to examine and connect those that already exist. The results of our analysis highlight this trend, as they show a growing number of reviews and studies of various kinds conducted on the Coronavirus phenomenon. The results examined refer to 81 scientific documents (Table 1), published between 2020 and 2023, which are considered to be quite indicative in order to address and answer the questions on which this research focuses: What is the current state of studies on CoViD-19 on adolescents, from 2020 to today? What are the themes most often addressed in studies on adolescence from psycho-physical, and learning point of view? What are possible suggestions for future research directions?

**Table 1:** Studies and research.

Study	Scope	Sample	Study design	Time and place	Results
Akdogan and Ergin (2022)	To study the effects of COVID-19	281 students from fourth to eighth	Quantitative	Istanbul, during the Covid-19 pandemic	52% of respondents reported having positively experienced restrictions imposed by COVID-19 (greater family commitment, more free time, more time for study…). 64% of respondents reported having negatively experienced restrictions imposed by COVID-19 (isolation, loneliness, anxiety…).
Toker (2022)	To investigate learning during the COVID-19 pandemic in urban and non-urban areas, where connectivity and access to technology could be limited.	4,501 eighth-grade students from urban and non-urban areas	Quantitative	Turkey, during the COVID-19 pandemic	The data showed a loss of learning for a year and a half both in Turkish and in mathematics, especially for girls. The data also shows that the family’s educational status helps children have fewer learning losses
Davidovitch et al. (2022)	To examine the influence of family functioning, and students’ motivation to study during the period of COVID-19.	148 young people aged 12–18	Quantitative	Israel, during the pandemic	Children of divorced parents have lower intrinsic motivation than children of married parents. This is one variable that most influences motivation to study and learning.
Feng et al. (2021)	To analyze the difference in student performance before and after the COVID-19 pandemic.	5,675 students in Grade 1 and Grade 9	Quantitative	China, during the COVID-19 pandemic.	The primary findings indicate that student performance before the pandemic was significantly better than after the pandemic. Online teaching has had a higher negative effect in rural areas compared to urban ones.
Lane et al. (2021)	To determine the impact of the COVID pandemic 19 on the anxiety of students.	2,990 French-Canadian students of junior high school (Grade 7) and high school (Grade 8)	Quantitative descriptive	Québec, before and after the COVID-19 pandemic	The pandemic has had variable impacts on the mental health of students. Some reported negative effects on their lives, others reported no effect and some have reported positive effects. However, the students interviewed during the pandemic have reported symptoms of generalized anxiety and high levels of test anxiety, fear of judgment and perfectionism, compared to those interviewed before the pandemic.
Cheong et al. (2023)	Examines intrapersonal characteristics or factors (peer relationships, resilience…) and family processes (e.g., parental involvement, critical confrontation) as potential risk and protective factors for adolescent mental health during COVID-19.	504 middle school students	Quantitative unstructured	Beijing, September 2020.	Personal resilience and quality of peer relationship represented two factors that predicted positive mental health. Additionally, children reported experiencing various stressful life events related to COVID-19.
Montreuil et al. (2023)	To examine how public health measures, implemented during the COVID-19 pandemic, have affected the mental health of children and adolescents.	25 children and adolescents aged 6–17	Qualitative	Canada, before and after the pandemic	Negative impacts on the mental health of children and adolescents have been linked to the loss of social activities, imposed public health measures, the shift to online learning and challenges with family relationships. Furthermore, some youngsters have shared positive reflections on the pandemic context.
Comelli et al. (2021)	To explorie the students’ perspective on distance education during the COVID-19 pandemic	Adolescents aged 11–17	Qualitative, with online questionnaire	Brazil, during the COVID-19 pandemic	During this study, four affective fields were identified: friends, class, home, and teachers. These affective fields allowed the authors to consider the triad friends- family-teachers, considered as fundamental for overcoming the emotional tiredness identified during the survey. Students expressed negative emotions about remote teaching.
Stone et al. (2021)	To evaluate sleep–wake times for 1–2 weeks in a group of adolescents who have experienced the transition from in-person learning to distance learning.	Adolescents of average age 12	Quantitative descriptive	Australia, during the Covid-19 pandemic.	The results demonstrate that during distance learning, lesson times were postponed. This ensured an extension of rest times, which in turn had a positive benefit on adolescents.
Roche et al. (2022)	To examine the impact of the Coronavirus (COVID-19) pandemic on the adaptation of Latinx adolescents.	547 adolescents of average age 13.5	Quantitative descriptive	Latin America, during the COVID-19 pandemic.	Child and adolescent care due to COVID-19 has led to a significant increase in mental health symptoms and decline in academic performance among adolescents in Latin America.
Li et al. (2022)	To assess the psychological impact and lifestyle of Australian adolescents, during the COVID-19 pandemic.	760 Australian adolescents aged 12–18	Qualitative	Australia, during the pandemic.	Three quarters of the sample have experienced worsening mental health since the start of the pandemic, with important negative implications reported on learning, friendships, and family relationships. Compared to normative samples, the data also show high levels of sleep disturbance, psychological distress, and health anxiety.
Villanti et al. (2022)	To examine the impact of COVID-19 restrictions on mental health and substance abuse in young people.	Adolescents aged 12–17, young adults aged 18–25	Quantitative descriptive	Vermont, during the Covid-19 pandemic.	Over 60% of participants noted that they had experienced negative effects from the pandemic on their physical, emotional, and social well-being. The impact of stressors related to COVID-19 was greater in young adults than in adolescents. This has resulted in young people becoming more involved in substance abuse (tobacco, cannabis…).
Schwartz et al. (2021)	To analyze the experiences of adolescents after returning to school.	2.310 students aged 12–18	Quantitative descriptive	Canada, after the end of the pandemic.	The first results show that on average young people managed to adapt well to returning to school, but the survey revealed that some subgroups need additional support.
Perkins et al. (2021)	To study the correlation between distance learning and anxiety-depressive symptoms on a group of teenagers during the Covid-19 pandemic.	320 students of middle and high school	Mixed (quantitative with online survey)	Massachusetts, during the pandemic.	Results demonstrate a correlation between distance learning and students’ mental health during the time of COVID-19.
Ye et al. (2022)	To examine learning disabilities and cyberbullying cases among a group of Chinese adolescents. To assess to what extent the student–teacher relationship can moderate any negative situations.	733 middle school students	Qualitative	China, during the Covid-19 pandemic.	Results suggested that perceived online learning difficulties and cyberbullying predicted greater mental health difficulties; perceived difficulties with online learning predicted academic commitment negatively. The student-teacher relationship also moderated the relationship between cyberbullying and mental health, as well as difficulties with online learning and academic engagement.
Ashworth et al. (2022)	To explore the experiences and impact of Covid-19 restrictions on pre-adolescents, in particular the coping strategies that may have helped adolescents during isolation.	Adolescents aged 11–14	Mixed	England, during the COVID-19 pandemic.	The results show that many viewed the initial period positively, as they had more free time and could dedicate themselves to other hobbies (cooking, art, gaming and watching television). However, over time they began to lose relationships with their peers.
Hawes et al. (2022)	To investigate the state of depression and anxiety in adolescents during the COVID-19 period.	451 adolescents and young adults	Quantitative	United States, during the pandemic	In one of the early epicenters of the COVID-19 pandemic in the United States, adolescents and young adults have experienced increased symptoms of depression and anxiety. Pandemic-related school and home confinement concerns were independently associated with changes in symptoms. Overall, this report suggests that the COVID-19 pandemic is having multiple negative effects on mental health of young people.
Gray (2020)	To investigate the effects of COVID-19 on children and adolescents	Children and adolescents aged 8–13.	Qualitative	United States, in the first months of the COVID-19 pandemic.	The findings demonstrate that increased time for play, greater opportunity to contribute constructively to family life, and greater family togetherness improved the mental well-being of many children during the first months of the pandemic.
Raby et al. (2021)	To assess the educational impact of the pandemic n children and adolescents in Ontario	Children and adolescents aged 8–15	Qualitative	Ontario, in the early stages of the COVID-19 pandemic.	The results demonstrate that children and adolescents received different support depending on their geographical location and family origin. These two variables had different impacts on both children and adolescents.
Layman et al. (2023)	To evaluate the association between sources of social support (parents and school) and adolescents’ perceptions of the emotional impact related to COVID-19 on their mental health	Adolescents (average age 11)	Quantitative	North America, during the Covid-19 pandemic.	Both parental and school social support have been associated with better mental health outcomes. Nonetheless, the impacts of COVID-19 on the lives of adolescents have been rated negatively in terms of mental and emotional health.
Salmela-Aro et al. (2021)	To examine school engagement and burnout profiles among adolescents before and during COVID-19. To study latent change within the classroom and stability in students’ social–emotional skills	1,381 students of Grade 5 and 6 and 1,374 of Grade 7 and 8	Quantitative descriptive	Finland, during the Covid-19 pandemic.	The current findings have showed the importance of social–emotional skills in promoting students’ academic well-being. To better support students at risk of burnout during the pandemic, school counselors could help students create coping strategies for dealing with stressors.
Boruchowicz et al. (2021)	To study the effects of the COVID-19 pandemic on school and work in young adults.	Adolescents aged 12–18	Quantitative	Mexico, during the COVID-19 pandemic.	The results demonstrate a 30% reduction in total hours dedicated to studies, for those who declared they spent at least 1 h on studies. The time dedicated to work showed, however, fewer variations compared to the time dedicated to study.
Swords et al. (2021)	To explore changes in adolescents’ lives during the initial transition to distance learning in the USA.	Adolescents of average age 13.5	Quantitative	United States, during the early stages of the COVID-19 pandemic.	The results demonstrate that, during the first wave, the restrictions and the transition to distance learning were not experienced negatively by adolescents.
Liu et al. (2021)	This study aims to identify the correlation between depression/anxiety and the COVID-19 pandemic lockdown, in children and adolescents after the easing of restrictions.	5,175 Chinese children and adolescents	Quantitative	China, after the COVID-19 pandemic.	The results showed that 12.33 and 6.26% of all participants reported depression and anxiety after the lockdown.
Julianto et al. (2023)	This study aimed to determine the mental health condition of vocational high school students in Indonesia during the pandemic.	Adolescents aged 10–12	Quantitative	Indonesia, during the Covid-19 pandemic.	The results showed that more than 60% of Indonesian vocational students experienced mental health problems during the COVID-19 pandemic due to social restrictions and online learning. Furthermore, the results of this study showed that mental health problems were mostly experienced by female students, firstborns, and students living in rural areas and from middle-income backgrounds.
Kirsch and Vaiouli (2023)	The present study examined adolescents’ perceptions of schoolwork while learning from home and at school.	Adolescents aged 12–16	Qualitative	Luxembourg, during the COVID-19 pandemic.	The results show that participants perceived their work as less interesting/useful and more difficult while learning from home.
Silk et al. (2022)	To examine risk and protective factors for health and emotional problems in adolescent girls during the COVID-19 pandemic.	American adolescent young girls aged 12–17 years.	Quantitative	United States, during the COVID-19 pandemic.	Girls reported engaging in many activities that can contribute to well-being. The analysis of mixed effects revealed positive impacts associated with better emotional health, such as more time for family and relaxation and less pressure from school/activities. Negative impacts were associated with poorer emotional health and included problems with online schooling, lack of space/privacy, lack of a regular schedule and family conflicts.
Zhang et al. (2022)	To study the relationship between Internet addiction and aggressive behavior, in an attempt to identify the mediating effects of depression and anxiety.	1,148 middle school students	Quantitative	China, during the COVID-19 pandemic.	The results suggest that (1) there was a significant positive correlation between Internet addiction and aggressive behavior; (2) anxiety, but not depression, mediated the effect of Internet addiction on aggressive behavior; (3) gender did not moderate the effect of Internet addiction on aggressive behavior.
Saini et al. (2023)	To study the experiences, and perceived impact, of COVID-19 among early adolescents and their parents.	Adolescents aged 11–13 and their parents	Qualitative	England, during the COVID-19 pandemic.	Parents and adolescents were realistic about the situation and helped each other; they put mental health and happiness first, not by comparing themselves with others, but by entertaining each other; followed guidelines/kept themselves safe; they enjoyed their time together, doing exercise/activities physically, playing together, talking and taking a break from work.
Zhou et al. (2023)	The present study aims to examine the associations between sedentary behavior and negative emotions, and also tries to investigate whether social support and the quality of sleep mediated this relationship.	1,179 middle and high school students	Quantitative	China, during the time of COVID-19.	The results of the present study suggest that social support and sleep quality partially mediate the relationship between sedentary behavior and negative emotions in middle and high school students during home confinement.
Bertoletti et al. (2023)	Investigates the heterogeneous impact of school closures during the COVID-19 pandemic in Italy on the academic performance of different groups of students.	508 schools in Grade 5 (primary) and 390 schools in Grade 8 (middle).	Quantitative	Italy, during the COVID-19 pandemic.	The results confirm that learning loss was considerable, although heterogeneity exists between disciplines and classes.
Conto et al. (2021)	School disruption and dropout impact children’s and adolescents’ acquisition of key skills before and during the COVID-19 pandemic.	Children and adolescents aged 9–14	Quantitative	UNESCO, during the COVID-19 pandemic.	Countries’ distance learning responses are often inadequate to keep all children learning, avoid school dropouts and mitigate learning losses.
Eales et al. (2021)	To examine the use of social media from the point of view of parents on their children before and during the COVID-19 pandemic.	Children and adolescents aged 2–13	Mixed	United States, during the COVID-19 pandemic.	The increase in addiction to screens has been influenced by distal, proximal and maintenance factors including the COVID-19 pandemic, distance learning, children’s behaviors, other children, the parental mediation and positive reinforcement by the media.
Xia et al. (2022)	This study investigated ruminative thinking and social support as mediating variables to explore the influence of negative life events on depression during the period of COVID-19 pandemic.	493 middle school students	Quantitative	China, during the COVID-19 pandemic.	Ruminative thinking and perceived social support had a significant mediating ripple effect on adolescent life events and depression.
Bonal and Gonzalez (2020)	This article evaluates the impact of school lockdown on the learning gap between children and adolescents from different social backgrounds.	Children and adolescents aged 3–18	Qualitative	Catalunya, during the COVID-19 pandemic.	Middle-class families were able to maintain higher standards in terms of quality of education in a critical context, while children from socially disadvantaged families had few learning opportunities both in terms of time and learning experiences. The results differed by type of school (public/private), economic, social and cultural capital of the family and its living conditions.
Ionescu et al. (2020)	The document analyzes the sustainability of the e-learning system implemented in Romania during the pandemic.	Teachers-students (of all grades and orders) and parents	Quantitative	Romania, during the COVID-19 pandemic.	The research findings indicate that students have accepted online learning, even though they find it less attractive than the traditional education system.
Schmiedeberg and Thonnissen (2021)	To investigate how, in addition to situational factors such as employment, family status and health, personality traits can influence how individuals experienced the initial crisis.	9,640 subjects, including some adolescents	Qualitative	Germany, in the early stages of the COVID-19 pandemic.	Personality plays a fundamental role in individual perceptions of the pandemic situation. Furthermore, the results show that the majority of people perceived not only negative, but also positive aspects of the COVID-19 pandemic situation in 2020.
Zhao et al. (2022)	School closures and home confinement due to the COVID-19 pandemic can lead to sleep disorders. As a result, the risk of mental disorders in children and adolescents might increase.	Children and adolescents (aged 6 and 17)	Quantitative	China, during the COVID-19 pandemic.	Out of a total of 873 participants (19.9%), 1,100 participants (25.1%) and 670 participants (15.3%) reported symptoms of depression, anxiety, and stress, respectively. Significant changes in both sleep duration and sleep–wake cycle patterns were observed before and during the COVID-19 pandemic.
Liu et al. (2022)	This study aims to investigate the direct and indirect relationships between technology and school engagement through psychopathological symptoms (i.e., depression, anxiety, insomnia) in early, middle and late adolescence.	4,852 adolescents	Quantitative	China, during the COVID-19 pandemic.	Depression and insomnia -as well as anxiety and insomnia- mediated the relationship between technology use and academic engagement. Anxiety showed a double-edged effect, i.e., a positive relationship with academic engagement and a negative relationship with academic engagement through insomnia.
Shukla et al. (2022)	To explore adolescents’ resilience to COVID-19 pandemic stress and examine key aspects of well-being across all countries using network analysis.	Adolescents aged 12–18 and 12–25	Qualitative	India, Israel, and United Kingdom, during the COVID-19 pandemic.	The British and Israeli networks focused on “dealing with problems well,” while the Indian network focused on “feeling useful.”
Delvecchio et al. (2022)	To compare psychological symptoms and coping strategies during home confinement due to COVID-19.	1,480 children of preschool and school age and adolescents	Quantitative	Italy, Portugal, and Spain, during the COVID-19 pandemic.	The results showed that preschool children showed more difficulty sleeping, had tantrums and showed addiction, while adolescents’ reactions were more related to worries and the uncertainty of COVID-19.
Weingart et al. (2021)	To investigate sleep parameters and predictors in adolescents during school closures due to COVID-19.	Adolescents from middle and high school	Qualitative	United States, during the COVID-19 pandemic.	Students reported waking up 2.1–2.9 h later during school closures and slept an average of 7.9–8.7 h and 8.6–9.5 h during term time. Compared to middle school students, high school students went to bed and woke up later.
Bravo-Sanzana et al. (2022)	It aims to investigate the well-being of adolescents in Mexico and Chile during the pandemic and to explore the relationship between victimization and types of hedonic and eudemonic well-being.	Adolescents aged 10–18	Quantitative	Mexico and Chile, during the Covid-19 pandemic.	Although most adolescents in samples from both countries had thriving experiences, some belonged to groups with moderate mental health. This is especially evident in the late adolescent group of both countries.
Pillay (2023)	To identify the difficulties of South African adolescents during the COVID-19 pandemic.	4,230 students from Grade 4–12	Qualitative	South Africa, during the COVID-19 pandemic.	Students self-reported the difficulties they encountered during the most severe part of the COVID-19 lockdown period, which impacted their mental health. The results indicate that fear was the most experienced difficulty, but this must be read in comorbidity with anxiety, stress and depression.
Orgilés et al. (2020)	To examine for the first time the emotional impact of quarantine on children and adolescents from Italy and Spain.	1.143 parents of Italian and Spanish children aged 3–18 anni	Qualitative	Italy and Spain during the COVID-19 pandemic.	The results show that 85.7% of parents perceived changes in their children’s emotional state and behaviors during the quarantine. The most frequent symptoms were difficulty concentrating (76.6%), boredom (52%), irritability (39%), restlessness (38.8%), nervousness (38%), feelings of loneliness (31.3%), discomfort (30.4%) and worries (30.1%). Spanish parents reported more symptoms than Italian parents.
Asscheman et al. (2021)	They explored daily fluctuations in mood during the first months (April 2020–June 2020) of the COVID-19 pandemic. Furthermore, the role of parents and peers on variability of adolescents’ mood was considered.	54 adolescents and 132 parents	Qualitative	Netherlands, in the early stages of the COVID-19 pandemic.	Early adolescents experienced low levels of attachment to their parents or peers, and specifically felt a low connection with their parents or peers (i.e., high levels of alienation), and reported more fluctuations in their daily mood than the first teenagers that experienced a strong level of attachment to their parents or peers.
Jin et al. (2022)	To study the level of anxiety sensitivity and its risk factors in children and adolescents in Northwest China during the pandemic lockdown of COVID-19 in early 2020.	Students aged 9–17	Quantitative descriptive	China, during the COVID-19 pandemic	During the COVID-19 pandemic and home quarantine, scores measuring the prevalence of anxiety sensitivity in children and adolescents in northwest China were high.
Hallauer et al. (2021)	To examine relationships between established predictors of problematic cell phone use (depression and anxiety) and a potential mediator of problematic cell phone use (anxiety sensitivity).	4,752 middle and high school students	Quantitative	China, during the COVID-19 pandemic	Structural equation modeling results indicated that anxiety was positively associated with AS when adjusting for depression; and AS was significantly associated with greater severity of PSU, adjusting for age and sex. Furthermore, AS mediated the relationships between anxiety and severity of PSU.
Bernasco et al. (2021)	To examine whether internalizing problems during the pandemic could be predicted by friends’ support before the crisis and whether this effect was moderated by adolescents’ time spent with their friends and stress related to COVID-19.	Adolescents of average age 12	Quantitative descriptive	Netherlands, during the COVID-19 pandemic.	Greater friend support pre-COVID-19 predicted fewer internalizing problems (self-reported and parent-reported) during COVID-19, and this effect was not moderated by adolescents’ time spent with friends or COVID-19-related stress. Friends can therefore protect against the development of internalizing symptoms in times of crisis.
Alivernini et al. (2020)	To study the importance of the following factors (emotions, personality, and motivation) in predicting PDB (physical distancing behavior)	347 adolescents	Quantitative descriptive	Italy, during the COVID-19 pandemic.	After the national COVID-19 lockdown, adolescents experienced fewer positive emotions and more negative emotions than the year before. However, these emotional changes and adolescents’ personality (with the exception of openness to experiences) were not related to PDB adoption. Instead, adolescents’ autonomous motivation significantly predicted a greater likelihood of adopting PDB by increasing the intention to engage in this behavior and, more indirectly, by substantially decreasing moral disengagement, which was negatively related to PDB. In contrast, controlled motivation corresponded to significantly higher levels of moral disengagement and predicted less likelihood to adopt PDB.
Cooper et al. (2021)	To investigate the effect of loneliness, social contact, and relationships with parents on adolescent mental health during lockdown in the UK	Adolescents aged 11–16 anni.	Quantitative	United Kingdom, during the COVID-19 pandemic.	During lockdown, teenagers who felt lonely reported experiencing more mental health symptoms. Loneliness during lockdown was not associated with mental health symptoms a month later. Teens who felt close to their parents at the start of lockdown reported having fewer mental health symptoms 1 month later.
Duan et al. (2020)	This study aims to demonstrate the psychological effects on children and adolescents associated with the epidemic.	Children and adolescents aged 7–18	Quantitative	China, during the COVID-19 pandemic.	The presence of clinical depressive symptoms, residence in urban regions, the implementation of precautionary and control measures, being female, having a family member or a friend infected with coronavirus have been associated with increased levels of anxiety.
Valido et al. (2023)	In 2020, Sources of Strength launched an elementary school program focused on promoting protective factors and resilience with the goal of understanding whether disruptions associated with the COVID-19 pandemic have significant long-term impacts on a variety of youth outcomes.	Middle and high school students.	Quantitative	United States, during the COVID-19 pandemic.	The COVID-19 pandemic has had a huge impact on the mental health of students in the United States. Rates of stress, anxiety and depression in children skyrocketed when they transitioned to online learning in early 2020. These rates only increased as social isolation, fear of disease and economic turmoil continued into 2021.
Simoës-Perlant et al. (2022)	This study examined symptoms of exhaustion, school stress, and anxious school refusal from a comparative developmental perspective in French adolescents enrolled in general, technological and vocational schools, both public and private.	423 middle and high school students.	Qualitative	France, during the COVID-19 pandemic.	The data shows unprecedented results on the perception of school stress, school burnout and anxious school refusal in adolescents aged 10–19 and highlights a very high percentage of suffering among adolescents. Young people most affected are high school students, and more specifically those of 12th grade, regardless of sex or schooling.
Garthe et al. (2023)	The current study examined changes in cyber victimization and mental health symptoms from before and during the COVID-19 pandemic.	Pre-adolescents	Quantitative	United States, during the COVID-19 pandemic.	The current study highlights the importance of SEL and violence prevention programming in schools to reduce the likelihood of cyber-victimization and associated mental health outcomes.
Tao et al. (2023)	The present study systematically examined and compared networks of depressive symptoms across different populations.	36.105 participants from junior high school, high school, university and seniors.	Quantitative	China, during the COVID-19 pandemic.	The results showed that sad mood was the most prominent symptom among middle school, high school, and college students, but the most prominent symptom in older adults was guilt. Among the top three core symptoms, suicidal ideation was unique to high school students, while Anhedonia was more widespread among university students.
Borgonovi and Ferrara (2023)	We use longitudinal data from over 1.5 million Italian students to examine differences in achievement in mathematics and reading in those students who completed primary and lower secondary school in 2020–21 (COVID cohort) and those who completed it in 2018–19 (non-COVID cohort). We also examine the evolution of inequalities by gender, socio-economic status, and previous academic achievement during the pandemic.	Italian students of primary and junior high school	Quantitative descriptive	Italy, during the COVID-19 pandemic.	On average, the primary school COVID cohort saw a small increase in reading achievement and a decline in mathematics achievement compared to the non-COVID cohort. The COVID cohort of lower secondary school saw a large reduction in mathematics achievement and a smaller reduction in reading achievement compared to the non-COVID cohort. Previously, average-achieving students suffered most from the pandemic, while high-achieving students gained. Socioeconomic disparities in achievement remained stable for secondary school students but decreased somewhat for students of primary school between the non-COVID and COVID cohorts.
Han et al. (2023)	This study explored the effects of coping style and two potential intermediate factors (cognitive reappraisal and psychological resilience) on the mental health of middle school students during normalization of prevention and control of the epidemic in China.	743 students of middle school (386 boys, 357 girls, 241 Grade 6 students, 235 Grade 7 students and 267 Grade 8 students)	Quantitative	China, during the COVID-19 pandemic.	The results showed that coping style, cognitive reappraisal, and psychological resilience directly predicted mental health. The negative effect of a negative coping style on mental health was significantly stronger than the positive effect of a positive coping style. Coping style affected mental health through the independent mediating effects of cognitive revaluation and psychological resilience, and through their chain mediation.
Kim et al. (2023)	To study the effects of gaming during the COVID-19 pandemic on health-related risk behaviors.	Middle and high school students	Quantitative	China, during the COVID-19 pandemic.	Students who exhibited high levels of gaming use also exhibited high levels of health-related risk behaviors, with significant sex-related differences in the high-risk group and in risk behavior. The levels were particularly high in terms of stress, physical inactivity, depression, smoking, and fatigue.
Doz and Doz (2023)	This study examined whether students’ math anxiety (MA) levels assessed before and during the first wave of the pandemic changed as a result of implementing remote learning.	117 Italian middle and high school students.	Quantitative	Italy, in the early stages of the COVID-19 pandemic.	No significant differences were found between pre- and mid-pandemic MA when considering the entire sample. When analyzed separately, results indicated that students with high MA reported significantly lower levels of MA during distance learning, however no difference was observed for individuals with moderate and low MA.
Capurso et al. (2022)	This retrospective-descriptive study investigated how elementary and middle school children perceived the first COVID-19 lockdown in Italy (March–May 2020) through drawing.	Elementary and middle school children aged 7–13	Qualitative	Italy, in the early stages of the COVID-19 pandemic.	Most children used colorful, full-body representations of the self, but in nearly half of the pictures drawn by the older students, the self was missing or depicted without a visible face. Most children drew the inside of their homes, and the outside world was completely invisible in more than half of the pictures. The most represented activities among younger students were games or sports, followed by screen or the use of technology.
An et al. (2022)	To study and explore the impact of the attitude towards peer interaction on students’ mindset, including motivation to online learning and practicing critical thinking, which could influence their self-efficacy in solving problems during the COVID-19 pandemic.	Middle school students	Quantitative	China, during the COVID-19 pandemic.	It was found that attitude towards peer interaction could positively predict middle school students’ online learning motivation and critical thinking.
Pirrone et al. (2022)	The present study explores differences in students’ anxiety levels toward mathematics during remote or in-person schooling.	405 students, recruited from 12 middle schools in the province of Catania (Italy)	Quantitative	Italy, during the COVID-19 pandemic.	The results showed a lower state of anxiety experienced during distance learning. However, students who preferred to learn math in person revealed less math anxiety and better mental states and metacognitive awareness; the same results were found in those who reported higher grades in mathematics and who preferred scientific subjects. It seems that math anxiety is not one of several shortcomings that are attributed to distance learning.
Dias et al. (2022)	This exploratory study aims to analyze the directions taken in teaching activities in public and private schools in the city of Rio de Janeiro (Brazil) and their consequences for learning and academic performance in elementary and middle schools.	Children and adolescents from primary and middle schools	Quantitative	Brazil, during the pandemic.	Students and teachers had to adapt to a new way of teaching and social isolation that interfered with their emotions and feelings. Despite new and positive learning experiences, this led to some counterproductive consequences as it was imposed suddenly, without adequate time for those concerned to assimilate the new methods correctly The percentage of students who improved their academic and learning performance was low compared to the percentages of those who maintained or reduced the same performance.
Balayar and Langlais (2022)	To examine the determinants related to students’ learning performance before and during the COVID-19 period in association with psychosocial behaviors (such as socialization, behaviors of internalization ed. externalization and motivation) and other factors, including the support received from parents, the way of teaching, and access to digital resources.	80 parents of children from primary and middle schools	Qualitative	United States, during the COVID-19 pandemic.	The findings indicated that more than double the normal time was spent by parents supporting their children’s learning and development during the COVID-19 period. Such factors as parental support and motivation were found to be the most effective contributors to the development of children’s positive emotions and achievement of learning. It was indicated that academic performance, motivation to participate in learning, socialization, prosocial behavior, discipline, externalizing and internalizing behaviors decreased during the COVID-19 pandemic.
Swartz and Benz (2022)	This mixed-method study was set in a public middle school in the Pacific Northwest. Existing data on attendance and responses to a researcher-generated survey of students who met the state definition of chronic absenteeism were analyzed to explore changes in students’ self-reported feelings of connectedness to school, relationships with teachers, relationships between peers and school climate before the COVID 19 pandemic and during Comprehensive Distance Learning (CDL).	105 middle school students.	Mixed	North America, during the COVID-19 pandemic.	The findings suggest a decrease in how positive school relationships are formed, peer relationships are cultivated and maintained, and school climate is cultivated during Comprehensive Distance Learning (CDL). These changes have had a significant impact on the degree to which students feel connected to school in a virtual environment.
Borualogo and Casas (2022)	This paper examines the subjective well-being (SWB) of children and adolescents during the COVID-19 pandemic in Indonesia in two periods (May–July 2020 and March–May 2021).	Children and adolescents (aged 10–18)	Quantitative descriptive	Indonesia, during the COVID-19 pandemic.	The results showed that boys showed significantly higher mean SWB scores than girls, while the elementary students have showed significantly higher mean scores for the cognitive component than middle and high school students for both data collection periods. Boys also showed significantly higher PA scores than girls and grade differences on PA and NA, depending on the study period.
Xin et al. (2022)	This study explores the relationship between adolescents’ perceptions of epidemic risk and their emotions through three follow-up surveys during the early stages of the COVID-19 pandemic on the 11th (Q1), 18th (Q2), and 25th. (T3) February 2020.	304 adolescents of various school levels (junior high school, senior high school and university)	Quantitative descriptive	China, in the early stages of the COVID-19 pandemic.	The results found that the individual’s positive emotions were significantly higher than negative emotions at T1, T2, and T3. The cross-lagged analysis found that for positive emotions, T2 positive emotions could negatively predict the individual’s T3 epidemic risk perceptions, and T2 epidemic risk perceptions could negatively predict the individual’s T3 positive emotions. For negative emotions, risk perceptions at T1 could positively predict negative emotions at T2 and, at the same time, the negative emotions at T1 could also positively predict epidemic risk perceptions at T2. This indicates that, during the early stages of the COVID-19 pandemic, there was a causal relationship between epidemic risk perceptions and adolescents’ emotions, and this relationship had a high stability between different gender groups and school levels.
Haser et al. (2022)	The study explored the occurrence of the loss of mathematics learning among Turkish middle school students during COVID-19 school closures through the practices, challenges and efforts reported by mathematics teachers as they attempted to support their students’ learning.	Middle school students	Quantitative	Turkey, during the COVID-19 pandemic.	Reports from middle school mathematics teachers indicated that the main reasons for mathematics learning loss among Turkish middle school students were existing inequalities and students’ limited or lack of access to the teacher, learning environment, learning and teaching materials. Although teachers have developed practices to engage students in teaching and learning interaction, student participation, methods of teaching, family SES and involvement moderated the effects of inequalities and access to learning resources.
Aguilar et al. (2022)	How does live teaching relate to student involvement in distance learning? Does the relationship differ across grade levels? This study addresses these questions by examining data from a random sample of families in a large school urban district in Southern California.	Students from middle and high school and their families	Quantitative	California, during the COVID-19 pandemic.	Our findings support the hypothesis that live online instruction is important when engaging students during distance learning, which can lead to greater connection with teachers and peers. This is even more important in earlier grades, when students have yet to develop the skills required for independent learning.
Vira and Skoog (2021)	This study aimed to evaluate differences in the psychosocial well-being of Swedish students from before to during the pandemic.	Students from Swedish middle schools	Quantitative descriptive	Sweden, during the COVID-19 pandemic.	Based on this first longitudinal study on student well-being during the COVID-19 pandemic, Swedish middle school students who continued formal schooling show mainly positive adaptations, and therefore appear to be resilient during the COVID-19 pandemic.
Doz (2021)	The goal of this study is to address the differences between math grades before and after the COVID-19 pandemic and, specifically, investigate the possible differences between intermediate and final grades in Italy.	231 students from Italian middle and high schools.	Quantitative	Italy, during the COVID-19 pandemic.	The findings suggest that more caution should be exercised when interpreting students’ grades before and after the COVID-19 lockdown, as it cannot be ruled out that such students’ results are inflated.
Huang et al. (2021)	The goal is to examine the emotional states of middle school students in Wuhan during the COVID-19 pandemic from the students’ perspective only.	15 middle school students	Qualitative	China, during the COVID-19 pandemic.	Feelings of loss of control and negative emotions were commonly found in these students. Second, the emotional states of middle school students in Wuhan changed substantially at different time nodes during this pandemic. They felt frightened and afraid when the city was blocked; and these feelings were developed into feelings of loss of control and negative emotions. They became happy and excited when the lockdown was lifted.
Shen et al. (2021)	This study aims to examine the mediating role of mobile phone addiction in the relationship between PTSD and academic boredom.	631 middle school adolescents	Quantitative	China, during the COVID-19 pandemic.	Adolescents’ PTSD symptoms directly aggravated their academic boredom and indirectly affected academic boredom by increasing their addiction to cell phones.
Khlaif et al. (2021)	To study the impact of distance learning on students from fifth to ninth grade.	Middle school adolescents	Quantitative	Palestine, during the COVID-19 pandemic.	Based on the findings reported by participants, it appears that the COVID-19 crisis has negatively affected student engagement in emergency remote learning due to new challenges emerging during the learning process.
Yan et al. (2021)	This study explores how students at different stages of their primary and secondary education have responded to full-time mandatory online learning during the COVID-19 pandemic.	1.170.769 Chinese students	Quantitative	China, during the COVID-19 pandemic.	The findings identify year-round differences between K-12 students’ online learning experience during the COVID-19 pandemic.
Akbari (2021)	The present research analyzed the challenges and effectiveness of using SHAD social network in the COVID -19 era from the perspective of teachers, parents and middle school students in Tehran.	75 interviews among middle school teachers, parents and students	Qualitative	Iran, during the COVID-19 pandemic.	Noticeable differences between private and state schools in addressing problems and challenges. Participants felt that the quality of teaching in an online environment was lower than in-person teaching, which they considered preferable. The degree of learning has also declined in the COVID -19 era, although both teachers and students have devoted more time and energy to teaching and learning, and parents (in particular) have been significantly more engaged.
Lee J. et al. (2021)	The main aim of the present study was to explore how learning attitudes and risk perceptions towards COVID-19 influence poor academic performance, since the onset of the COVID-19 pandemic, among middle school students.	Middle school students	Quantitative	China, during the COVID-19 pandemic.	Because middle school students are not as mature as adults and the period during middle school is critical in development, they are more likely to be greatly impacted by the unprecedented COVID-19 pandemic. Therefore, it is important to understand changes in the academic performance of middle school students since the beginning of the COVID-19 pandemic. However, there is little evidence identifying what factors influence declines in student grades since the start of the COVID-19 pandemic. This study revealed that two factors -learning attitudes and risk perception towards COVID-19 were significantly associated with poor academic performance.
Liu et al. (2021)	This study aimed to identify related factors for the depression/anxiety among children and adolescents after the COVID-19 pandemic lockdown.	Children and adolescents	Qualitative	China, during the COVID-19 pandemic.	12.33 and 6.26% of all participants reported depression and anxiety post-lockdown, separately. Suicidal ideation, arguments with parents, insomnia, difficulty concentrating during online learning, and anxious and depressed mood during lockdown were positively associated with depression and anxiety after lockdown.
Stefanidou and Mandrikas (2023)	This paper examines the views of Year 6 and Year 10 students on the remote teaching of sciences during the COVID-19 pandemic in Greece.	Students of both primary and secondary school	Quantitative	Greece, during the COVID-19 pandemic.	Their opinions seemed to be negatively affected by technical issues such as poor network connection, limited student–student and student-teacher interactions and by the partial or complete lack of laboratory activities. However, a limited number of students reported some positive experiences in the extended period of distance learning. These positive aspects are the greater and effective dissemination of audiovisual material, such as selected software, animations, and videos.
Anyika et al. (2021)	This current study explored the impacts of the COVID-19 pandemic on Nigeria’s education system and in the process provided a distinctive solution to the challenges facing the sustainability of education in the country.	Middle school students and their teachers	Quantitative descriptive	Nigeria, during the COVID-19 pandemic.	Our study highlighted the importance of institutionalizing online distance education or web-based learning system in the Nigerian education system as the new normal in post-COVID-19 education. The study also identified that students’ engagement in e-education increases their competence and confidence in acquiring digital knowledge and skills and mastering how to apply them in their learning activities. On the part of teachers, teaching with smart devices promotes creativity and immense digital literacy to educate students effectively and enhance skill-based learning.

The scientific works examined for the creation of the aforementioned scientific review are based on studies conducted mainly in the psychological and social fields. They have an exploratory-experimental character, given that the authors’ interest has been focused on delving deeper into the theme of the psychological, social, and academic influences that the COVID-19 pandemic has had on the lives of adolescents (11–13 years old), especially from a psychological and learning perspective.

In this case, the research in question aims to confirm or disconfirm the hypothesis that the spread of the Coronavirus and the measures adopted to combat it have had negative repercussions on the mental well-being of adolescents and on their educational learning, following the sudden transition to distance learning.

To analyze the consequences of the pandemic phenomenon on their psychological well-being and on school learning, the samples in these works involved the participation of adolescents (11–13 aged) and, consequently, middle school students. This was because the authors intend to understand whether the adolescents (11–13 aged) who have lived through this period have suffered any negative implications, from a psychological and educational learning perspective.

To provide an accurate response to the aforementioned study hypotheses, many authors have made use of questionnaires, self-report interviews, quantitative measurement tools and scales, to arrive at an objective confirmation of the results obtained from both a both qualitative and quantitative perspective.

The results of the same research highlight that the pandemic has led to psychological disorders of various kinds on most of the subjects under study, a clear decline in learning and has fueled social inequalities, in terms of poor availability of electronic devices and tools and low connectivity. At the same time, a smaller number of studies capture the positive side of distance learning, highlighting the need for its institutionalization, since web-based teaching can increase children’s digital skills and abilities and mastery of their use in learning activities (Anyika et al., 2021).

Our results should be interpreted with some limitations of the present study in mind. We used the Preferred Reporting Items for Systematic Reviews (PRISMA) methodology to identify the psychological effects and social impact of the pandemic exclusively on adolescents aged between 11 and 13, thus excluding studies that did not specify the exact age of the sample under examination and which did not focus on the psycho-physical and psycho-social effects and the social impact of the pandemic. Furthermore, when accounting for several possible confounding factors suggested in the literature, studies that did not specify the research protocols were considered ineligible. Therefore, the results cannot be generalized, but they can be a contribution to the development of new areas of investigation and research.

## Conclusion

Despite the above-mentioned limitations, we believe that this study makes an innovative and effective contribution to the literature, as it provides concrete data on possible mental health disorders among adolescents resulting from the spread of the Coronavirus.

Assessing psychophysical well-being is essential to providing guidelines to reduce the impact of quarantine on adolescents, who are at a critical age, and prevent the long-term psychological consequences of COVID-19 and related restrictions.

After all, adolescents were seen as more worried than preschoolers, angrier than schoolchildren, and the most fearful of COVID-19 infection. Middle school students showed greater difficulty concentrating, were more worried about COVID-19 infection and were more easily alarmed than preschoolers and schoolchildren and argued more frequently with the rest of the family than young students (14–18) (Delvecchio et al., 2022). The complex picture reported in our study underlines the importance of considering the age of subjects when evaluating their psychological reactions to COVID-19 and related resolution strategies.

As our research shows, the COVID-19 outbreak has had a significant psychosocial impact on adolescents. The findings on current levels of anxiety and depression not only highlight the need to address emotional distress, but also provide researchers with the scientific foundation to formulate targeted interventions based on significant influencing factors.

Regarding the schooling aspect, despite schools’ efforts to maintain learning activities during the lockdown, our analysis reflects significant inequalities, depending on family characteristics (income, education level) and school characteristics (level of education, school sector). Distance learning has also interrupted those orientation and accompaniment processes that are particularly important for adolescent students in study, work and life transitions. Furthermore, the digital divide and visible differences in access to technological devices among students have left some young people without the ability to connect to learning for at least 3 months or, more likely, 6 months (Bonal and Gonzalez, 2020).

## Data availability statement

The original contributions presented in the study are included in the article/supplementary material, further inquiries can be directed to the corresponding author.

## Author contributions

GM: Writing – original draft, Writing – review & editing, Data curation, Formal analysis, Supervision. MS: Writing – original draft, Writing – review & editing. FF: Writing – original draft, Writing – review & editing. LV: Data curation, Formal analysis, Supervision, Writing – review & editing.
